# Total pulmonary arterial reconstruction in a patient with arterial tortuosity syndrome affecting the pulmonary artery: a case report and review of the literature

**DOI:** 10.1186/s13019-024-02905-6

**Published:** 2024-07-10

**Authors:** Fahad M. Alshair, Amal S. Alsulami, Mohammad S. Shihata, Osman O. Alradi, Ragab S. Debis, Abdullah H. Baghaffar, Mazin A. Fatani

**Affiliations:** 1 Division of Cardiac Surgery, Department of Surgery, King Abdulaziz University Hospital, P.O. Box: 80215, Jeddah, 21589 Saudi Arabia; 2 Faculty of Medicine, King Abdulaziz University, Jeddah, Saudi Arabia; 3 Cardiothoracic Surgery Department, King Faisal Specialist Hospital and Research Centre, Jeddah, Saudi Arabia; 4 Cardiothoracic Surgery Department, Al‑Azhar University, Cairo, Egypt

**Keywords:** Arterial tortuosity syndrome, ATS, Pulmonary artery stenosis, Case report

## Abstract

**Background:**

Arterial tortuosity syndrome is a rare Autosomal recessive disease that leads to a loss of function of the connective tissues of the body, this happens due to a mutation in the solute carrier family 2 member 10 (SLC2A10) gene. ATS is more likely to occur in Large and medium-sized arteries including the aorta and pulmonary arteries. This syndrome causes the arteries to be elongated and tortuous, This tortuosity disturbs the blood circulation resulting in stenosis and lack of blood flow to organs and this chronic turbulent flow increases the risk of aneurysm development, dissection and ischemic events.

**Case presentation:**

A 2 years old Arabian female child was diagnosed with ATS affecting the pulmonary arteries as a newborn, underwent a pulmonary arterial surgical reconstruction at the age of 2 years old due to the development of pulmonary artery stenosis with left pulmonary artery having a peak gradient of 73 mmHg with a peak velocity of 4.3 m/s and the right pulmonary artery having a peak gradient of 46 mmHg with a peak velocity of 3.4 m/s causing right ventricular hypertension. After surgical repair the left pulmonary artery has a peak pressure gradient of 20 mmHg, with the right pulmonary artery having a peak pressure gradient of 20 mmHg.

**Conclusion:**

ATS is a rare genetic condition that affects the great arteries especially the pulmonary arteries causing stenotic and tortuous vessels that may be central branches or distal peripheral branches that leads to severe right ventricular dysfunction and hypertension. We believe that surgical treatment provides the optimum outcomes when compared to transcather approaches especially when the peripheral arteries are involved. Some challenges and hiccups might occur, especially lung reperfusion injury that needs to be diagnosed and treated accordingly.

**Supplementary Information:**

The online version contains supplementary material available at 10.1186/s13019-024-02905-6.

## Introduction

Arterial tortuosity syndrome (ATS) is a rare autosomal recessive disorder that causes the connective tissues in the body to lose function. It is caused by a mutation in the solute carrier family 2 member 10 (SLC2A10) gene which encodes the GLUT-10 protein, responsible for transporting ascorbate, a key component in connective tissue metabolism, serving as a cofactor for collagen and elastin production processes [1–4].

ATS occurs mostly in Large and medium-sized arteries. This syndrome causes the arteries to be elongated and tortuous, causing a disturbed blood flow, increased risk of atherosclerosis, persistent hypertension, aneurysms, dissections, ischemic events and cerebrovascular accidents [1–3]. Cardiac manifestations affecting patients with ATS include: ventricular hypertrophy, valvular regurgitation and atrial fibrillation [5, 6]. In addition to aortic and pulmonary arteries, systemic arteries such as may be affected as well [7–9].

ATS is a rare disorder with a limited number of reports discussing ATS, With even fewer reports about its effects on the great arteries and the surgical repair techniques. We believe that this case report will bring insight to this rare disorder and allow surgeons to appreciate the presentation, approach to management from the surgical point of view and how to manage these patients in a multidisciplinary setting.

## Case presentation

A 2 years old Arabian female child was diagnosed with pulmonary artery stenosis (PAS) as a newborn when an echocardiogram was performed in neonatal intensive care unit (NICU) when she was admitted as a case of meconium aspiration. She was transferred to our facility for further assessment.

The child’s medical history was significant for atonic seizures on Levetiracetam 60 mg/kg twice daily and Sodium Valproate 34.5 mg/kg twice daily and were stopped 8 months prior to surgery due cessation of seizures. Family history was significant for consanguinity (parents are first cousins) with similarly affected cousins from the paternal side in a male cousin (3 years of age) with a similar picture of tortuous great vessels requiring balloon dilatation, Raising suspicion of an autosomal recessive inheritance. Physical examination revealed: generalized joint hypermobility with Beighton score of 6, A weight of 11.6 kg at the 11th percentile (-1.25-SD), A height of 90 cm at the 33rd percentile (-0.43-SD), With a head circumference: measuring 45.5 cm at the 4th percentile (-1.79SD), Dysmorphic features which include a long face, large ears with a simple helix and mild micrognathia. A soft systolic murmur was heard over the left parasternal border. The Clinical picture was consistent and supports the clinical diagnosis of ATS.

**Fig. 1:**
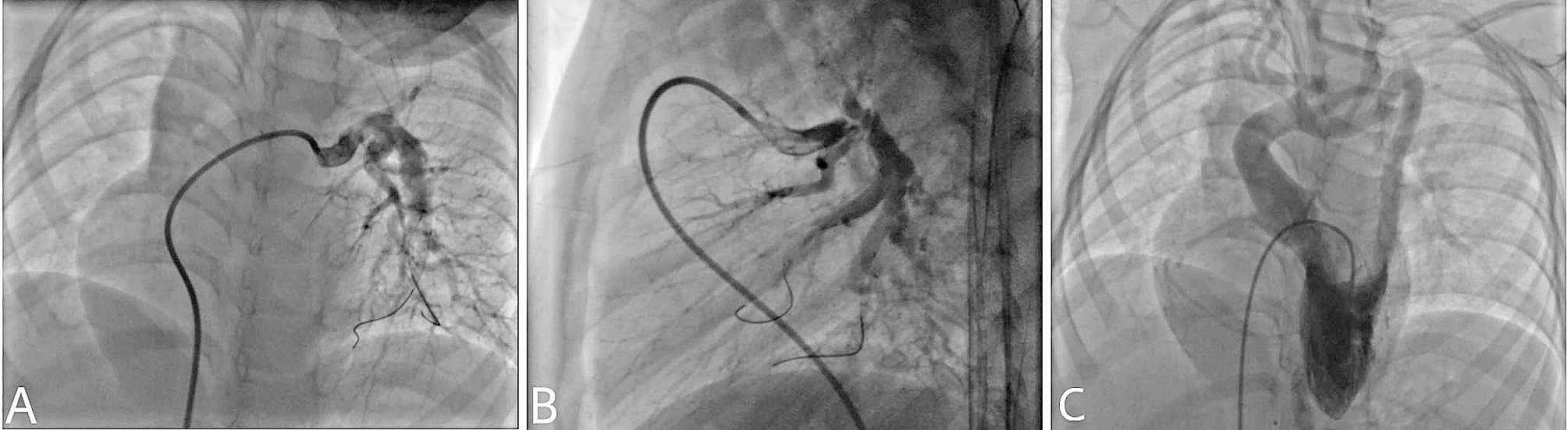
Preoperative cardiac catheterization and angiography. Preoperative cardiac catheterization and angiography): (**A**) demonstrating the anatomy of the tortuous left pulmonary arteries and it branches (**B**) demonstrating the anatomy of the tortuous right pulmonary artery and it branches (**C**) aortic angiogram revealing a tortuous aorta with no significant stenosis or structure. Cardiac catheterization revealed a right ventricular pressure of 136 mmHg with a RVSP/LVSP ratio of 1.2 mmHg. (see supplementary video 1)

A diagnostic catheterization was performed to assess the pulmonary tree and ascertain the extent of the tortuosity. Revealed a dilated main pulmonary artery (MPA), A tortuous left and right pulmonary arteries (LPA, RPA) with severe stenosis, A suprasystemic right ventricular pressure measuring 136 mmHg with a right ventricular systolic pressure left/ventricular systolic pressure ratio (RVSP/LVSP) of 1.2 mmHg. Angiography revealed a highly tortuous aorta with absence of any focal stenosis (Fig. 1).

**Fig. 2:**
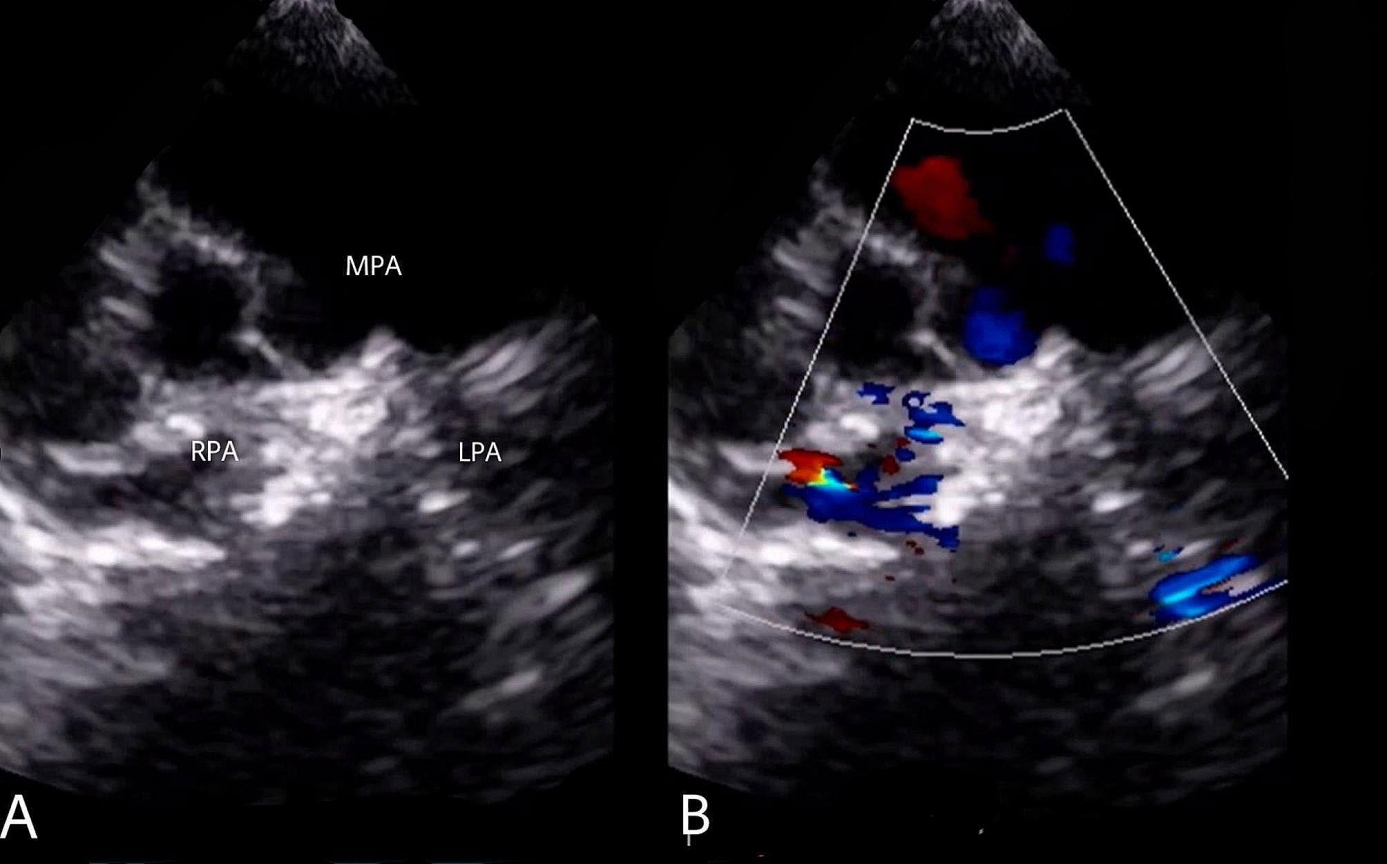
Preoperative transthoracic echocardiography. Preoperative transthoracic echocardiography (short axis view): (**A**) demonstrating the anatomy of the pulmonary arteries with an enlarged main pulmonary artery with small, tortuous and hypoplastic branch pulmonary arteries. (**B**) color flow doppler demonstrated that the left pulmonary artery has a peak gradient of 73 mmHg with a peak velocity of 4.3 m/s and the right pulmonary artery having a peak gradient of 46 mmHg with a peak velocity of 3.4 m/s. *(MPA: main pulmonary artery, RPA: right pulmonary artery, LPA: left pulmonary artery)* (see supplementary video 1)

Echocardiography measured the LPA to have a peak gradient of 73 mmHg and a peak velocity of 4.3 m/s and the RPA to have a peak gradient of 46 mmHg and a peak velocity of 3.4 m/s (Fig. 2).

The patient was operated on at the age of 2 years. The operation commenced with a standard median sternotomy. The Pericardium was opened and harvested carefully as it will be used to reconstruct the pulmonary arteries. Cardiopulmonary bypass was initiated through an aortic arterial cannula and a right atrial venous cannula, The procedure was performed on a beating heart with mild hypothermia. Dissection was performed to free the PAs up to the hila A patent ductus arteriosus was found; it was then ligated and resected. The MPA was clamped and snares were placed on the peripheral branches of the LPA and RPA.

**Fig. 3:**
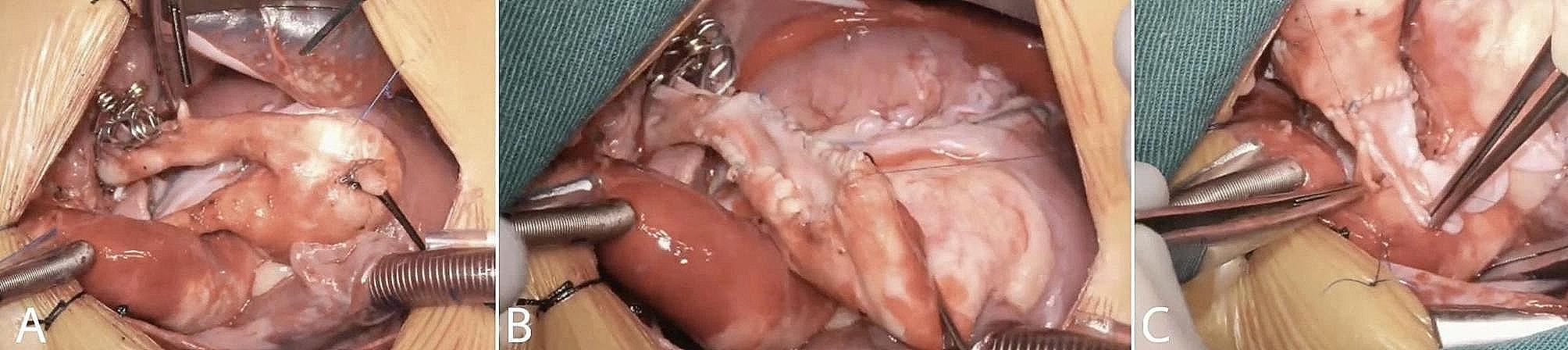
Intraoperative findings. Intraoperative findings: (**A**) anatomy of pulmonary artery after meticulous dissection revealing a large main pulmonary artery with small, hypoplastic and tortuous right and left pulmonary arteries. (**B**) repair of the left pulmonary artery after resection of the tortuous stenotic segment, using an arteriotomy incision and repair the defect using harvested autologous pericardium (**C**) repair of the right pulmonary artery with the same technique used for the left pulmonary artery. (see supplementary video 2)

The repair of the PAs was then commenced starting with the LPA An arteriotomy incision was performed to enlarged the stenotic artery and the autologous pericardial patch that was harvest was then fashioned and placed on the distal part of the arteriotomy incision, A continuous suture line was commenced till the point of the tortuosity which was then excised and an end-to-end anastomosis was performed to repair the defect from the excised part to the LPA. Then the pericardial patch’s continuous suture line was completed till it reached the proximal part of the arteriotomy. And the RPA was repaired using the same approach (Fig. 3). Cardiopulmonary bypass was weaned off without any difficulty with a bypass time of 99 min and transferred with minimal inotropic support which was weaned off the same day of surgery. It’s important to note that the patient did not develop lung reperfusion injury and didn’t require prolonged mechanical ventilation or the need for extracorporeal membrane oxygenator support or nitric oxide and was only managed with diuretics therapy.

**Fig. 4:**
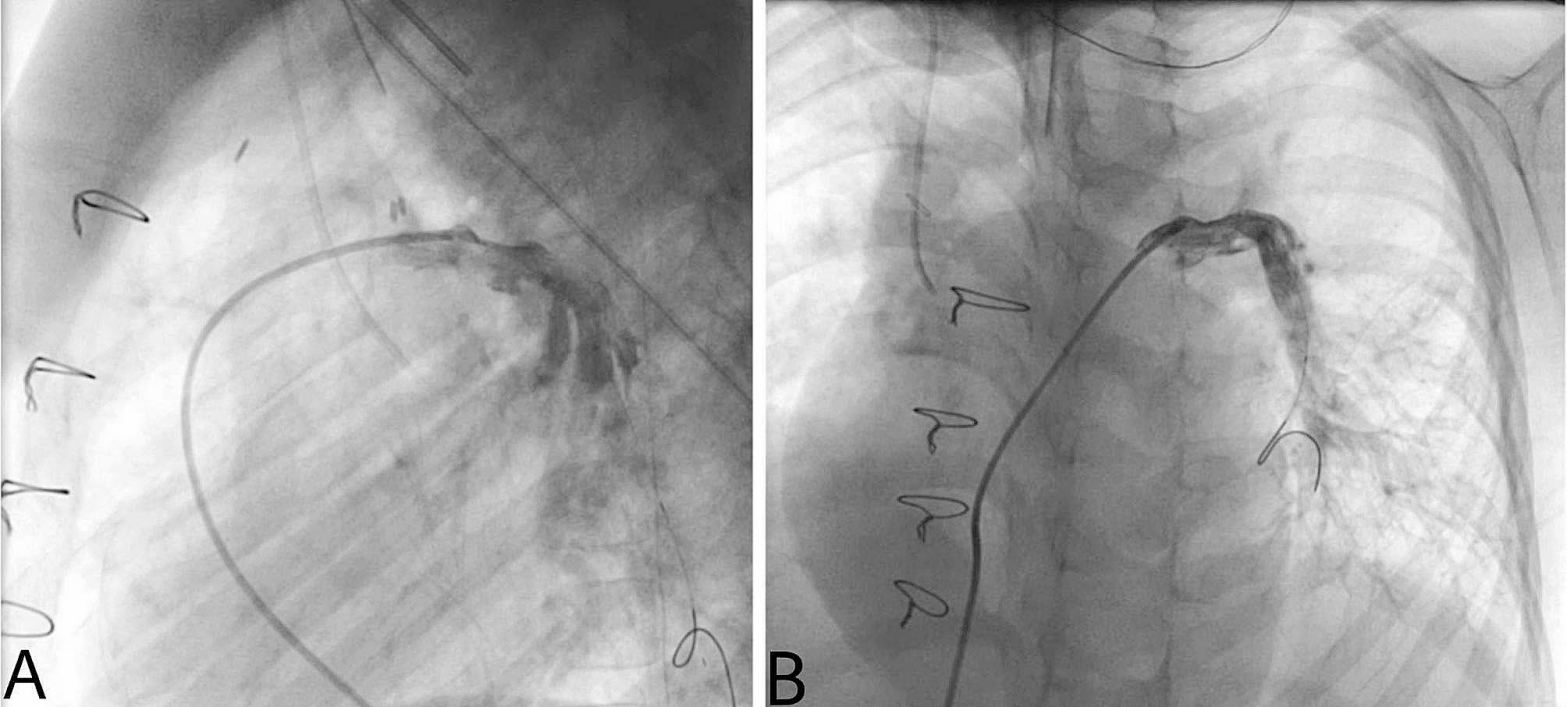
Postoperative cardiac catheterization and angiography. Postoperative cardiac catheterization and angiography): (**A**) demonstrating the anatomy of the right pulmonary artery after repair (**B**) demonstrating the anatomy of the left pulmonary artery after repair and ballooning which dropped the pressure to 14 mmHg. The right ventricular systolic pressure (RVSP) was 38 mmHg after repair. (see supplementary video 1)

A postoperative catheterization revealed significant proximal LPA origin stenosis with peak pressure gradient difference of about 25 mmHg, which was successfully dilated using a 8 × 40 mm Sterling Balloon (Boston Scientific, Marlborough, MA, USA) dropping the peak pressure gradient difference to about 14 mmHg. The right ventricular systolic pressure (RVSP) was 38 mmHg and the PA pressure was 38/15 mmHg with a mean of 24 mmHg (Fig. 4). The patient was discharged on postoperative day 7.

**Fig. 5:**
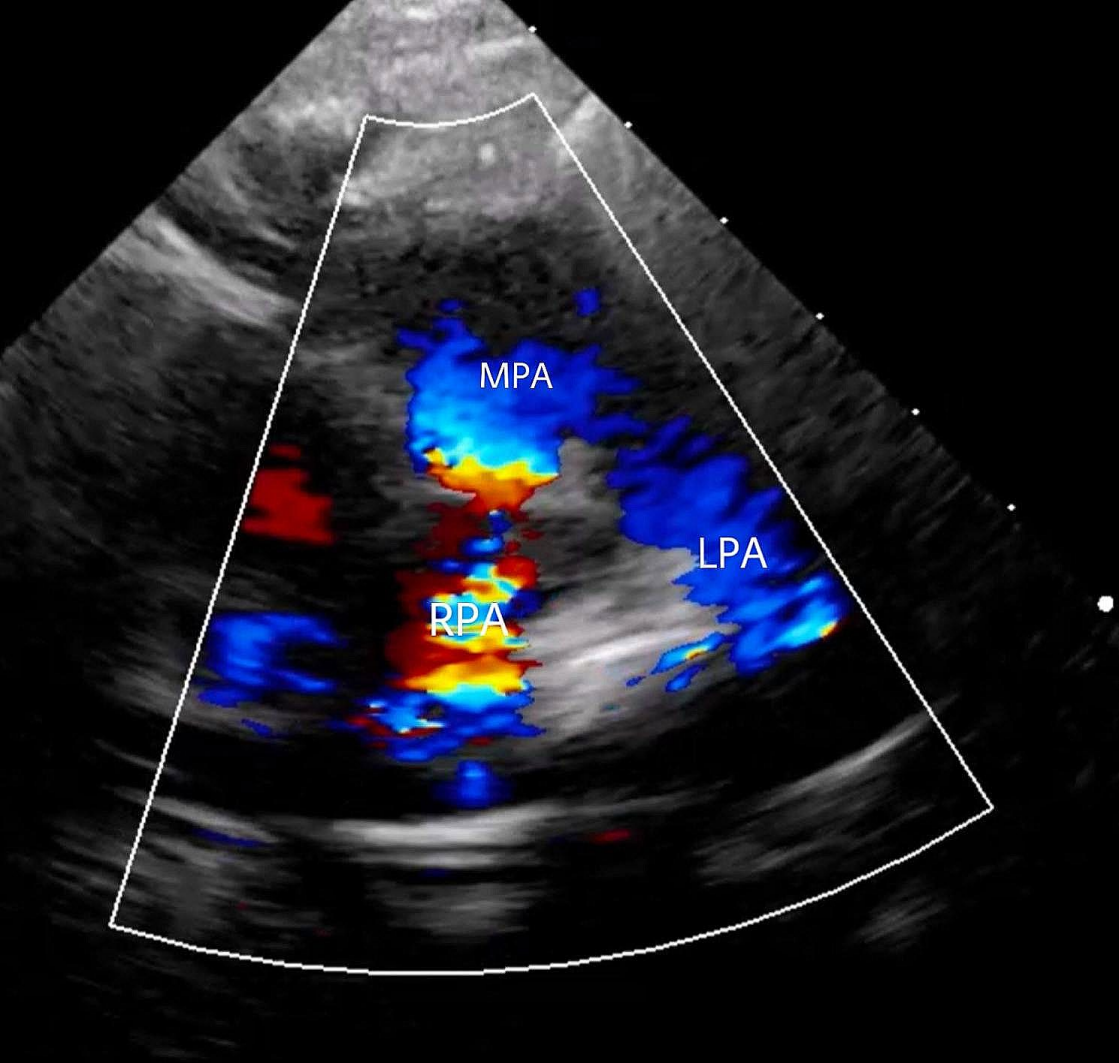
Postoperative transthoracic echocardiography. Postoperative transthoracic echocardiography (short axis view): demonstrating the anatomy of the pulmonary arteries with patent branch right and left pulmonary arteries. With the left pulmonary artery having a peak pressure gradient of 20 mmHg, with mild acceleration of flow across the right pulmonary artery with peak pressure gradient of 20 mmHg *(MPA: main pulmonary artery, RPA: right pulmonary artery, LPA: left pulmonary artery)* (see supplementary video 1)

**Fig. 6:**
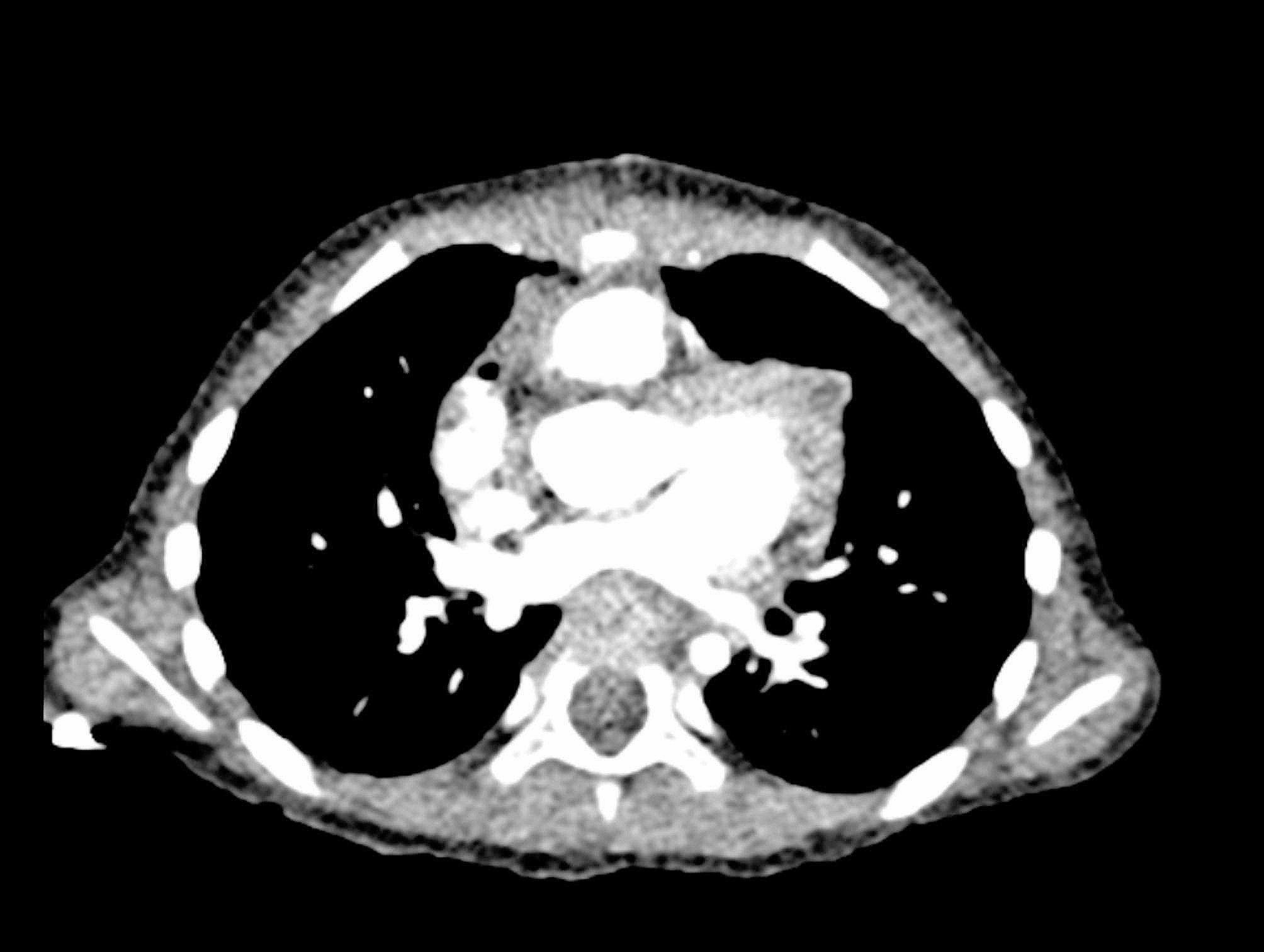
Postoperative computed tomography scan of the pulmonary arteries. Postoperative computed tomography angiography scan: showing patent main, left and right pulmonary arteries. (see supplementary video 1)

Follow up echocardiography at 2 months revealed a mild distal LPA stenosis with peak pressure gradient of 20 mmHg and a mild acceleration of flow across the RPA stenosis with a peak pressure gradient of 20 mmHg (Fig. 5). A postoperative computed tomography angiography of the PAs showed patent PAs (Fig. 6).

## Discussion

The earliest reports of ATS in the literature were by Ertugrul and Beuren and were labeled as a unique subset of Ehlers-Danlos Syndrome due to the similarities in the musculoskeletal features. It’s now Recognized as an autosomal recessive disorder with a mutation in the SLC2A10 gene which encodes the GLUT-10 protein, A key cofactor in the production of collagen and elastin [1, 2, 4, 7, 10–13].

Initial studies reflected an unfavorable prognosis with a high mortality rate of 40% prior to the age of five [5], later studies reported gentler outcomes [1, 2, 4].

ATS is more likely to occur in Large and medium-sized arteries including the aorta and PAs. It’s recommended to evaluate patients with echocardiography for the development of aortic root aneurysm every 3 months until 5 years, later on screening can be individualized but it should always be done annually [1, 2, 5].

The Incidence of peripheral PAS in patients with ATS varied between 21.9% [12], 28% [2] and 60% [5]. It results in right ventricular outflow obstruction with subsequent right ventricular hypertension, hypertrophy, dilation, and dysfunction. Presenting clinically with systemic venous congestion caused by an elevation in right atrial pressure, reduced exercise tolerance due to the inability to increase the cardiac output and in some cases cyanosis caused by a right to left shunt or in severe cases a very limited pulmonary blood flow [14].

To assess and classify the degree of PAS the RVSP/LVSP ratio can be used to address the physiologic impact of the disease and is useful to guide the need for intervention and assess its success. From the surgical and technical perspective, an anatomic classification of PAS is more appropriate. Multiple classifications have been suggested but the ideal classification describing the anatomic stenotic sites and burden which should represent the complexity of the repair which may predict the prognosis of the surgical intervention outcomes and allow comparison of various treatment strategies. A classification that fits this criteria was suggested by alkhaldi et al., where types 1 and 2 are considered “central PAS” as they are mostly in the intrapericardial segments and do not involve the intrapulmonary arteries, while types 3, 4, and 5 are considered “peripheral PAS” as they are intraparenchymal. Type 1: Involves the MPA with no extension to the ostia of the branches, Type 2: involves the branch PAs with no extension to the ostia of the lobar arteries, Type 3: Involves the lobar arteries with no extension to the ostia of the segmental arteries, Type 4: Involves the segmental arteries with no extension to the ostia of the sub-segmental arteries, Type 5: Involves the sub-segmental arteries or more distally [14–16]. The higher the classifications type was the more challenging the operation and repair were. With the mean cardiopulmonary bypass time, the number of angioplasties performed and the length of ICU stay increasing as the type increases.

The management of PAS in patients with ATS can be performed by either a surgical or a transcatheter approach with some cases describing a hybrid approach of both methods. The surgical approach can be performed using a single stage or a two stage approach, the first stage is done through a median sternotomy with the second stage through a left thoracotomy. The two stage approach is performed if the LPA distal branches were involved with severe and extensive stenosis. In the first stage the RPA is repaired through a median sternotomy, the LPA is repaired in the second stage through a left thoracotomy incision which allows excellent exposure. The timing of the second stage is performed during the same admission and it’s of great importance to allow the right lung to completely recover from lung reperfusion injury as the second stage is performed off pump which usually take 5 to 10 days but it may take longer if a patient required extracorporeal membrane oxygenation support [17].

The recommended reconstruction technique includes excising the stenotic tortuous segment and then anastomosing the defect created followed by an atriotomy to enlarge the PAs and a patch used to reconstruct the defect. The operation is performed while the patient is on cardiopulmonary bypass with a beating heart using mild hypothermia with a clamp occluding the main pulmonary artery and snares on the branch pulmonary arteries. The materials that can be used to reconstruct the pulmonary arteries include: bovine pericardium, pulmonary homografts, untreated or treated autologous pericardium, and the autologous excise pulmonary artery tissue. We prefer the use of a pulmonary homograft but it wasn’t used in our case due to the lack of supply and we didn’t use the excised pulmonary tissue as it wasn’t large enough to fit and completely reconstruct atriotomy.

In addition to surgical management catheter directed interventions can be used as well, mainly balloon dilation and stent placement in the tortuous stenotic segments. There are no studies looking at their use in patients with ATS but there are a plethora of large studies performed to assess their outcomes in patients without ATS with unsatisfactory outcomes showing an increased rate of recurrence and frequent need for catheter re-interventions [18].

In the largest studies on the topic, catheter interventions decreased the median right ventricular systolic pressure / left ventricular systolic pressure ratio by from 1.00 to 0.88. The mortality rate was 14% but only 7% resulted from the catheter intervention. Complications occurred in 16.6% of patients who required stenting. Balloon dilation had a 21% failure rate. 19% of patients required surgery with the majority occurring after a year from the interventions. Freedom from any re-intervention was only 38+/-6% at 1 year and 22+/-6% at 5 years. These unsatisfactory outcomes can be attributed to the fact that the peripheral pulmonary arteries are elastic leading to recoil resulting in the resistance to balloon dilation. Stenting isn’t advisable as well because of the widespread narrowing bilaterally, increasing the chance of blockage further down the artery after stenting because the stent would push the extra length of the tortuous wall forward [19–22].

There was a single case published using a hybrid approach management (Table 1); this approach was performed because imaging revealed that the stenosis was caused by redundancy of the proximal PAs, resulting in kinking and growth failure of their perihilar segments. This anatomical arrangement was deemed by the authors as unsuitable for surgical repair or simple angioplasty. They took the patient to the catheterization laboratory where a median sternotomy was performed. The stents were then introduced through an incision in the MPA and the LPA was dilated to 10 mm, The stenosis of the upper and lower branches of the RPA were dilated to 5 mm and 7 mm respectively. After that the patient was taken to the operating room where a V-shaped segment was resected from the inner circumference of the MPA branches and the defect was closed continuously. The outcomes of this procedure resulted in a decrease of the RVSP/LVSP ratio from 1.1 to 0.6 [23].

**Table 1:** Repaired pulmonary artery stenosis cases in patients with arterial tortuosity syndrome

Author (Year)	Type of paper	Number of patients	Age	Type of repair	Pre-operative hemodynamics	Post-operative hemodynamics	Lung reperfusion injury	Need for Catheterization re-intervention	Follow up period
Santoro et al., (2008) [23]	Case report	One patient	3 years	Hybrid (direct trans-catheter stenting + surgical repair)	RVSP/LVSP = 1.1	RVSP/LVSP = 0.6	No	No	6 months
Ricciardi et al., (2020) [22]	Case report	One patient	4 years	Surgical repair	RV = 96/3 mmHg, proximal – distal RPA = 84/26–29/12 mmHg, proximal LPA = 92/28 mmHg	RV = 50/-2 mmHg, proximal – distal RPA = 46/6–14/8 mmHg proximal LPA = 47/7 mmHg	No	No	5 months
Alkhaldi et al. (2022) [17]	Retrospective cohort	33	3 years (median)	Surgical repair (17 done in single stage, 16 done in dual stage)	RVSP/LVSP = 1.2 (mean)	RVSP/LVSP = 0.27+/-0.05 (mean)	Yes(to a variable degree)	No	1 year

*RV: right ventricle, RPA: Right pulmonary artery, LPA: Left pulmonary artery, RVSP/LVSP: Right ventricular systolic pressure/left ventricular systolic pressure*

A common complication and a challenge to manage after PAS surgery is pulmonary reperfusion injury. Its severity is associated with the extent of the pulmonary vascular disease. It can happen immediately after weaning of cardiopulmonary bypass or after 24 to 48 h. It usually manifests as increased bronchial secretions, bilateral lung infiltrates on chest radiographs and an increased alveolar-arterial oxygen gradient. Measures used to avoid and manage it should be started from the operating room with the use of continuous ultrafiltration on cardiopulmonary bypass to decrease tissue edema and total body water, after weaning nitric oxide can be initiated, postoperative measure can be performed which include: (1) diuresis, (2) keeping the hemoglobin at normal levels between 12 and 14 g/dL, (3) mechanical ventilation until the lung perfusion injury resolves with the use of a positive end-expiratory pressure (PEEP) of 4 to 5 cm H2O, (4) close observation for pulmonary hemorrhage, and (5) avoidance of aggressive endotracheal tube suctioning. Some patients might need a more aggressive approach to management with the use of extracorporeal membrane oxygenation for a few days to allow the lung to recover [17].

## Conclusion

ATS is a rare genetic condition that affects the great arteries especially the PAs causing stenotic and tortuous vessels that may be central or distal peripheral branches leading to severe right ventricular dysfunction and hypertension. We believe that surgical treatment provides optimum outcomes when compared to transcather approaches especially when the peripheral arteries are involved. Some challenges and hiccups might occur, especially lung reperfusion injury that needs to be diagnosed and treated accordingly.

### Electronic supplementary material

Below is the link to the electronic supplementary material.

Supplementary Material 1

Supplementary Material 2

## Data Availability

No datasets were generated or analysed during the current study.

## Abbreviations

ATS: Arterial tortuosity syndrome
RV: Right ventricle
PA: Pulmonary artery
MPA: Main pulmonary artery
RPA: Right pulmonary artery
LPA: Left pulmonary artery
RVSP/LVSP: Right ventricular systolic pressure / left ventricular systolic pressure
